# A Pediatric Case of Cubitus Varus Deformity due to Olecranon Fracture

**DOI:** 10.31662/jmaj.2023-0060

**Published:** 2023-09-25

**Authors:** Kazuya Yamada, Yuta Tsukagoshi, Toru Hoshi, Masazumi Suzuki, Yasuaki Hosono, Hayato Shimada, Shinsen Nomura, Yoshiyasu Ikezawa

**Affiliations:** 1 Department of Orthopaedic Surgery, Mito Saiseikai General Hospital, Ibaraki, Japan; 2 Department of Orthopaedic Surgery, Ibaraki Children’s Hospital, Ibaraki, Japan

**Keywords:** olecranon fracture, remodeling, corrective osteotomy, cubitus varus deformity, carrying angle, varus angulation, posterolateral rotatory instability

## Abstract

We encountered a pediatric case of cubitus varus deformity with a sheared olecranon fracture in an 8-year-old boy who underwent corrective osteotomy without relevant medical history. The patient fell, resulting in a sheared olecranon fracture. He underwent a closed reduction and casting. As the displacement slightly increased within a week, we followed him without secondary reduction to expect remodeling. No remodeling occurred; corrective osteotomy was performed one-year post-injury for a marked cubitus varus deformity. At 2.5 years after corrective osteotomy, little difference existed in the carrying angle (CA) and varus angulation (VA) of the proximal ulna than that of the contralateral side, without pain or limited range of motion.

The acceptable displacement range for pediatric forearm fractures is <1 cm shortening and 15° angular deformation in patients under 10 years old, and 10° angular deformation in older children. Here, the deformity of the ulna in the coronal plane did not remodel. Proximal forearm deformity can be accurately evaluated in flexion contracture elbows by measuring VA. Ulnar osteotomies are commonly performed on Monteggia fractures to reduce the radial head, and the osteotomy site is at the center of the deformity of the diaphysis. Corrective osteotomy for cubitus varus deformity after supracondylar humerus fracture improves function and cosmetic appearance, with good clinical results. In addition, it could prevent cubitus varus deformity from causing posterolateral rotatory instability.

The coronal-plane deformity of the proximal ulnar was not expected to remodel. We recommended early accurate reduction and consideration of additional internal fixation for preventing re-displacement. Corrective osteotomy for cubitus varus deformity of the proximal ulna was an effective treatment.

## Introduction

Proximal ulna fractures are common because the elbow lacks muscle or soft tissue protection. Olecranon fractures account for 4% of all pediatric elbow fractures and are sometimes associated with other ipsilateral elbow injuries resulting in poor outcomes ^(1)^. Pediatric cubitus varus deformity after supracondylar humerus fracture does not remodel ^(2)^. Since there are few reports of deformity in pediatric ulnar olecranon fractures, we report a pediatric case of cubitus varus deformity due to a sheared olecranon fracture requiring corrective osteotomy.

## Case Report

**Figure 1.**
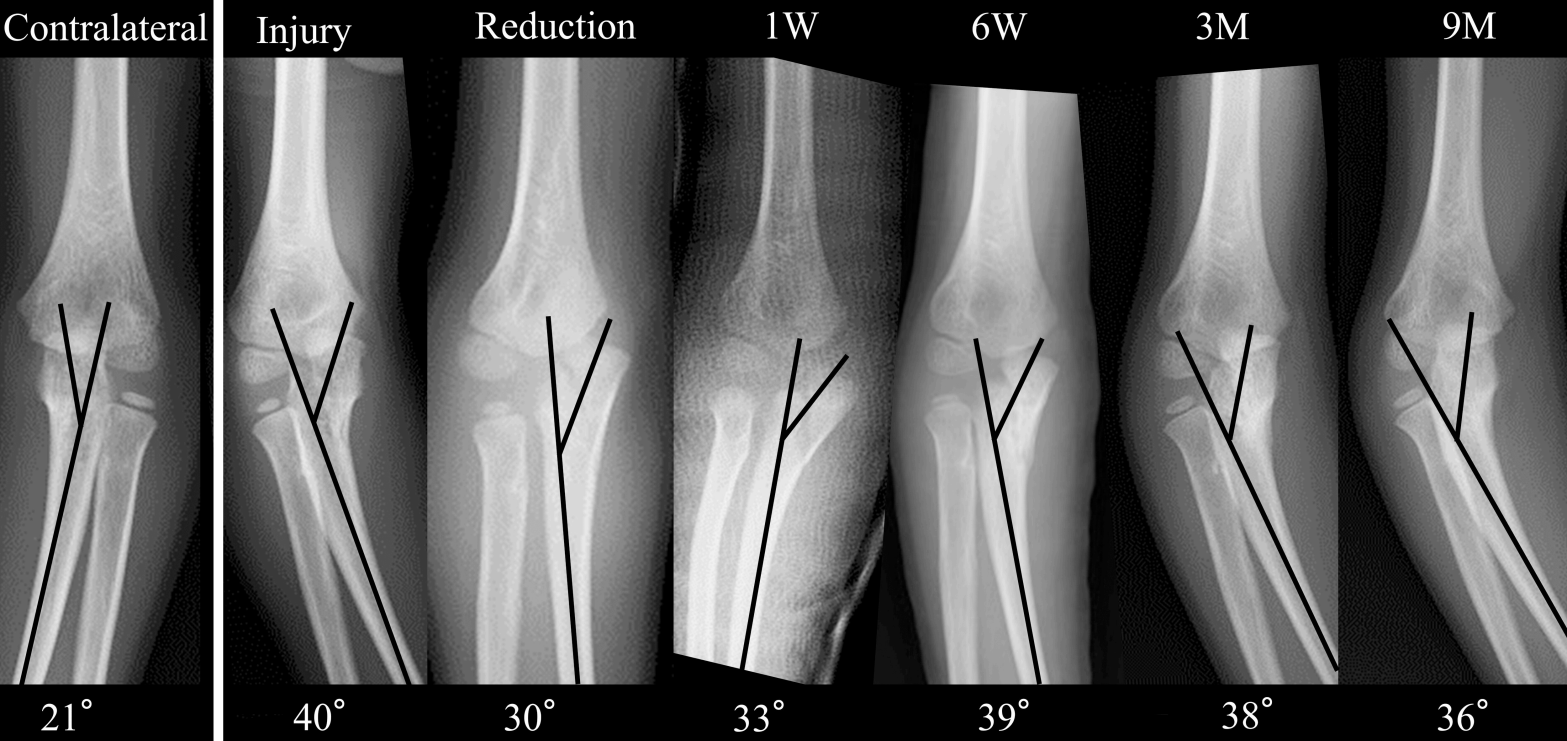
VA time lapse At the time of injury, the patient had an angulation of 19° relative to the contralateral side. The Reduction resulted in 9° of angulation, but at the end of the fixation period (6 weeks), the patient had 18° of angulation. VA, varus angulation.

**Figure 2.**
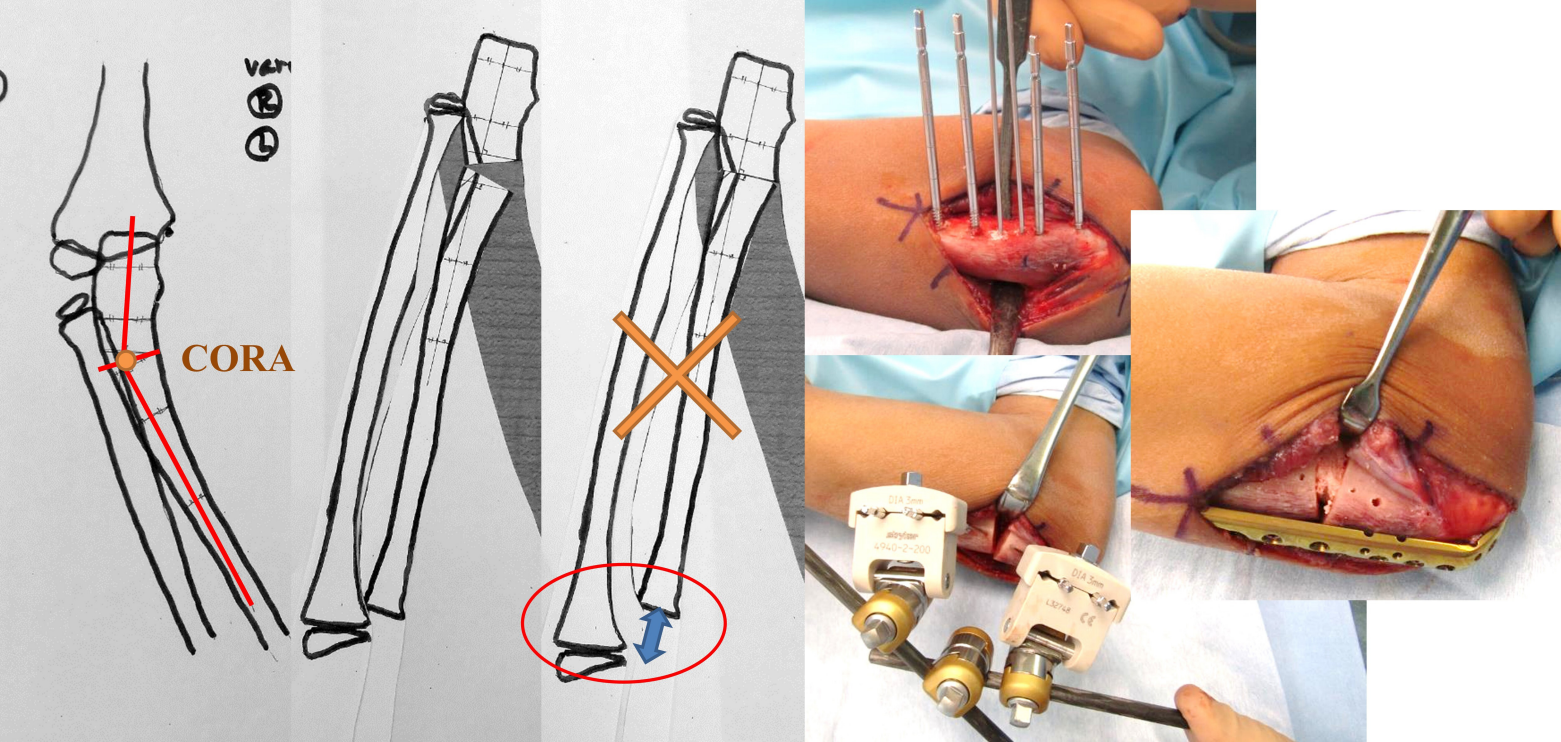
Preoperative planning and intraoperative macrophotography Osteotomy was performed at the center of rotation of angulation (CORA), slightly distal to the coronoid process of the ulna. An open wedge osteotomy was performed to correct the deformity considering the balance with the radial length. Plate fixation with artificial bone was planned. A corrective osteotomy of 30° was performed as planned while using external fixation.

**Figure 3.**
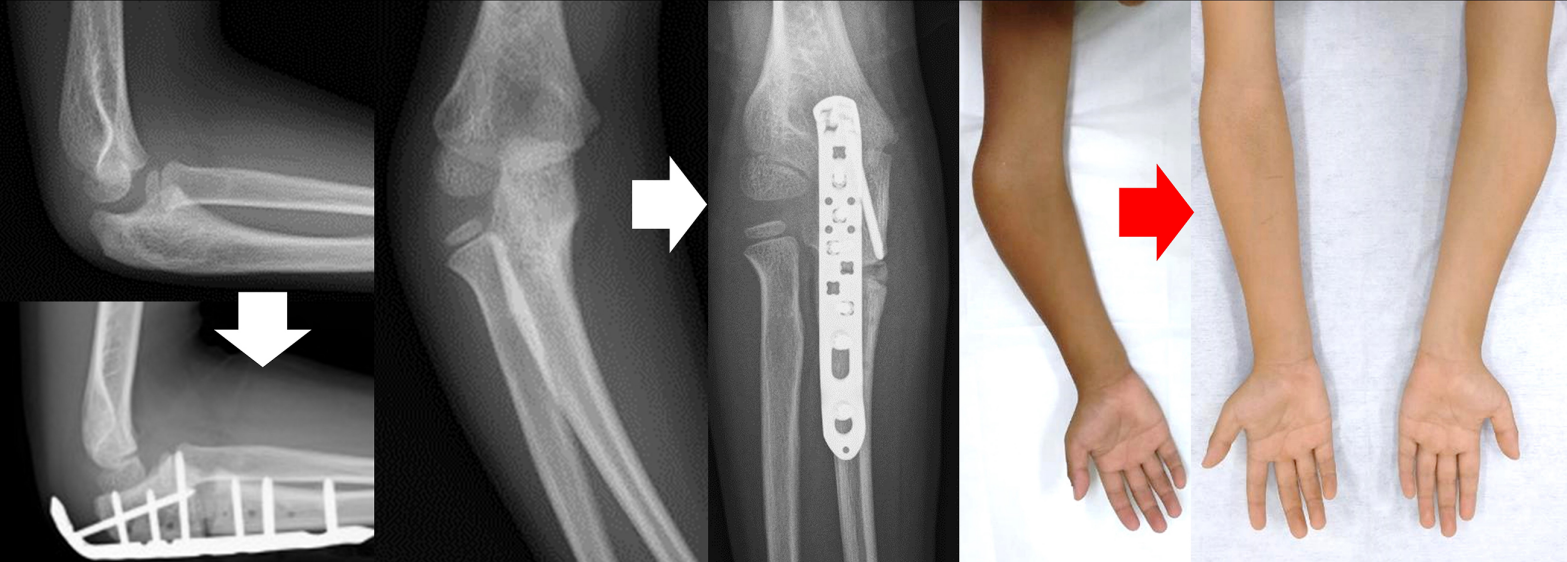
X-ray images and visual macrographs before and after corrective osteotomy Good alignment was maintained.

**Figure 4.**
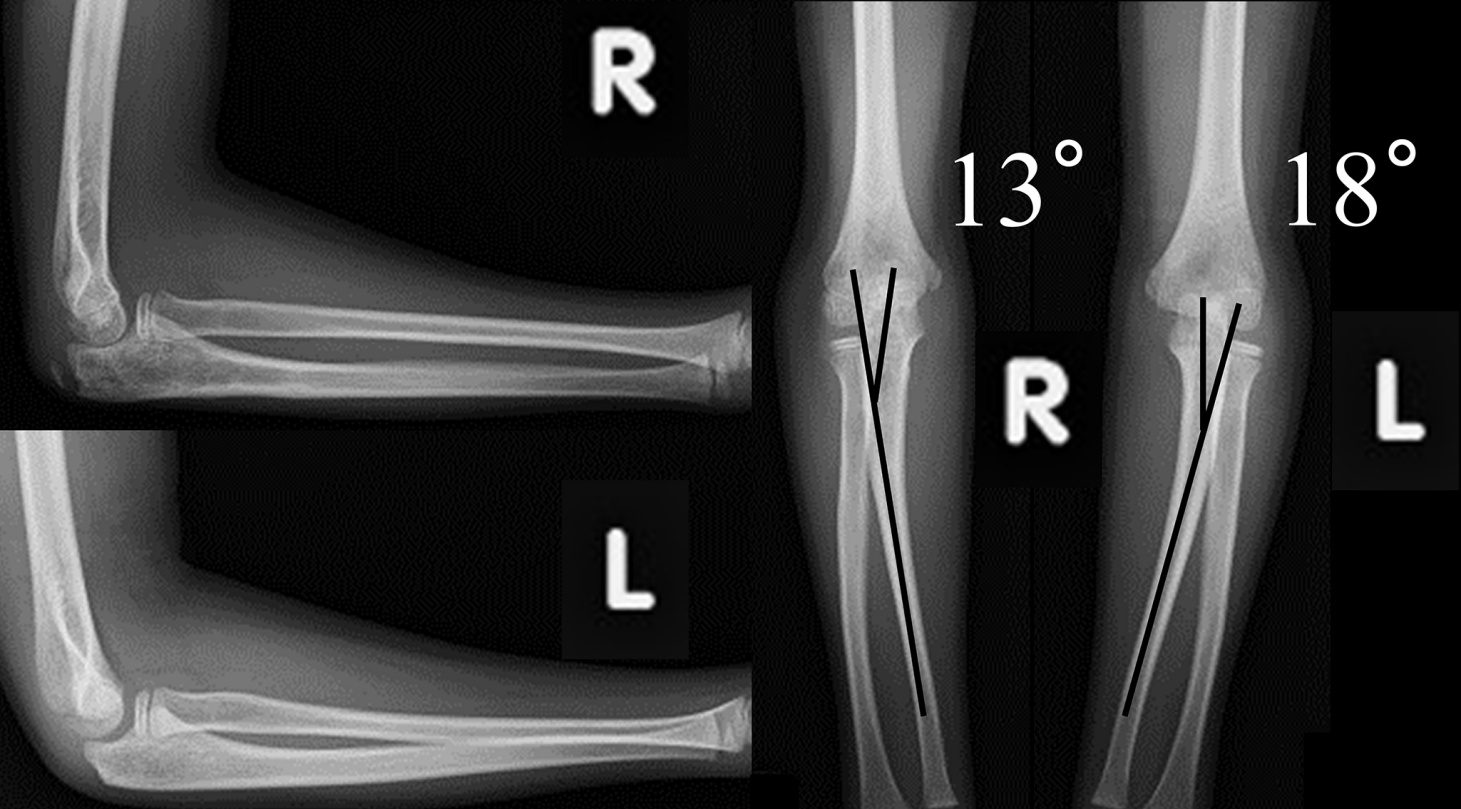
X-ray image at last follow-up The plate was extracted one year after the corrective osteotomy; the final follow-up was 3.5 years after the injury and 2.5 years after the corrective osteotomy. VA 13° (contralateral side 18°) and CA 10° varus (contralateral side 12° rotation) with little difference between right and left and no limitation of range of motion or pain. VA, varus angulation; CA, carrying angle.

An 8-year-old boy without relevant medical history fell, resulting in a sheared olecranon fracture. Displacement (5 mm), 24° of carrying angle (CA), and 40° of varus angulation (VA)^(3)^ were evaluated as proximal ulnar deformity, 12° and 21° on the contralateral side, respectively. On the day of injury, he underwent closed reduction under general anesthesia, and displacement was decreased to 2 mm, varus CA 4°, and VA 30°. Although immobilized in a cast, the elbow displacement increased to 4 mm, varus CA 7°, and VA 32° in one week after initial reduction, which we considered acceptable; he was followed without secondary reduction (Figure 1). Three months post-injury, with complete elbow extension, X-ray images showed varus CA 29° and VA 32°. No remodeling occurred during the one-year follow-up period. The radial head’s subluxation was palpable in rotation, although the range of flexion-extension and pronation-supination was full and painless. Corrective osteotomy was performed one-year post-injury to correct the elbow varus deformity (Figure 2 and 3). While using an external fixator, osteotomy was performed at the center of the deformity, slightly distal to the coronoid process of the ulna. The deformity was corrected with an open wedge to balance with the radial length, and artificial bone was inserted and fixed with a plate. The last follow-up was 3.5 years post-injury (2.5 years after corrective osteotomy), with varus CA 10° and VA 13°, 12° and 18° on the contralateral side, respectively, with slight bilateral differences in alignment, without pain or limited range of motion (Figure 4).

## Discussion

Regarding surgical indication for pediatric olecranon fractures, Holme recommended a displacement of >4 mm ^(4)^, and Wiley recommended one with the instability of the humeroradial joint ^(5)^. The acceptable displacement range for pediatric forearm fractures is <1 cm shortening and 15° angular deformation in patients under 10 years old, and 10° angular deformation in older children ^(6)^. Koh recommended anatomic reduction because persistent varus deformity of the proximal ulna causes subluxation and tilting of the radial head, resulting in radiocapitellar impingement ^(7)^. Here, although the angulation was within the indications range for conservative treatment, the coronal plane remodeling did not occur, necessitating deformity correction surgery. In elbow fractures, the joint cannot be fully extended owing to splint or pain, making accurate alignment evaluation difficult in the early post-injury period. An antero-posterior x-ray image of the elbow joint with flexion contracture may be more informative than the frontal view if the images are taken separately ― one aligned with the humerus and the other with the forearm. Grechenig defined VA as the medial angle of the 1/3 proximal ulna ^(3)^. Proximal forearm deformity can be accurately evaluated in flexion contracture elbows by measuring VA. Here, the VA after closed reduction was 30°, suggesting the initial reduction may have been inadequate.

Ulnar osteotomies commonly performed on Monteggia fractures reduce the radial head, and the osteotomy site is at the center of the deformity of the diaphysis. Corrective osteotomy for cubitus varus deformity after supracondylar humerus fracture is performed to improve function and cosmetic appearance, with good clinical results ^(8), (9)^. In cubitus varus, the mechanical axis, olecranon, and triceps axis are displaced medially. This causes repetitive cubitus external rotation that can stretch the complex lateral ligament and cause posterolateral rotatory instability ^(10)^. This case showed no functional impairment at one-year post-injury, but corrective osteotomy on the proximal ulnar deformity was performed to improve the appearance and prevent complications, such as the aforementioned instability over time. Internal fixation combined with intraoperative external fixation allowed us a quick and easy surgery by assessing the compatibility of the humeroradial joint, range of motion of rotation, and condition of the distal radioulnar joint before final internal fixation.

We encountered a pediatric case of cubitus varus deformity secondary to a sheared olecranon fracture. The proximal ulna coronal-plane deformity was not expected to remodel. Therefore, we recommended early accurate reduction and considered additional internal fixation to prevent re-displacement. Corrective osteotomy for cubitus varus deformity of the proximal ulna was effective.

## Article Information

### Conflicts of Interest

None

### Author Contributions

Kazuya Yamada: Substantial contributions to the conception and design of the work; the acquisition, analysis, and interpretation of data for the work; drafting the work; final approval of the version to be published; and agreement to be accountable for all aspects of the work in ensuring that questions related to the accuracy or integrity of any part of the work are appropriately investigated and resolved.

Yuta Tsukagoshi: Substantial contributions to the conception and design of the work; revising it critically for important intellectual content; final approval of the version to be published; and agreement to be accountable for all aspects of the work in ensuring that questions related to the accuracy or integrity of any part of the work are appropriately investigated and resolved.

Toru Hoshi: The acquisition of data for the work; drafting and revising it critically for important intellectual content; final approval of the version to be published.

Masazumi Suzuki: Final approval of the version to be published.

Yasuaki Hosono: Final approval of the version to be published.

Hayato Shimada: Final approval of the version to be published.

Shinsen Nomura: Final approval of the version to be published.

Yoshiyasu Ikezawa: Final approval of the version to be published.

### Approval by Institutional Review Board (IRB)

This case report did not require Ethical approval and consent because no additional tests or procedures were performed and was solely for research purposes. The disease was not an individually identifiable rare disease, and no human genome or genetic analysis was performed. Parental consent was obtained for using radiographs and clinical photographs included in this study.
